# Patterns of Medical Residents’ Preferences for Organizational Socialization Strategies to Facilitate Their Transitions: A Q-study

**DOI:** 10.5334/pme.1189

**Published:** 2024-03-11

**Authors:** Gerbrich Galema, Johanna Schönrock-Adema, Debbie A. D. C. Jaarsma, Götz J. K. G. Wietasch

**Affiliations:** 1 University of Groningen and University Medical Center Groningen (UMCG), and member of the Lifelong Learning, Education and Assessment Research Network (LEARN), She is also a resident in anesthesiology at the department of anesthesiology, NL; 2 Wenckebach Institute for Education and Training, University of Groningen and University Medical Center Groningen, and at the Prins Claus Conservatoire, Hanze University of Applied Sciences, Groningen, Furthermore, she is a LEARN member, NL; 3 Utrecht University, Faculty of Veterinary Medicine, and a LEARN member, NL; 4 University of Groningen and University Medical Center Groningen, Department of Anesthesiology, and a LEARN member, NL

## Abstract

**Introduction:**

To facilitate various transitions of medical residents, healthcare team members and departments may employ various organizational socialization strategies, including formal and informal onboarding methods. However, residents’ preferences for these organizational socialization strategies to ease their transition can vary. This study identifies patterns (viewpoints) in these preferences.

**Methods:**

Using Q-methodology, we asked a purposeful sample of early-career residents to rank a set of statements into a quasi-normal distributed grid. Statements were based on previous qualitative interviews and organizational socialization theory. Participants responded to the question, ‘What are your preferences regarding strategies other health care professionals, departments, or hospitals should use to optimize your next transition?’ Participants then explained their sorting choices in a post-sort questionnaire. We identified different viewpoints based on by-person (inverted) factor analysis and Varimax rotation. We interpreted the viewpoints using distinguishing and consensus statements, enriched by residents’ comments.

**Results:**

Fifty-one residents ranked 42 statements, among whom 36 residents displayed four distinct viewpoints: Dependent residents (n = 10) favored a task-oriented approach, clear guidance, and formal colleague relationships; Social Capitalizing residents (n = 9) preferred structure in the onboarding period and informal workplace social interactions; Autonomous residents (n = 12) prioritized a loosely structured onboarding period, independence, responsibility, and informal social interactions; and Development-oriented residents (n = 5) desired a balanced onboarding period that allowed independence, exploration, and development.

**Discussion:**

This identification of four viewpoints highlights the inadequacy of one-size-fits-all approaches to resident transition. Healthcare professionals and departments should tailor their socialization strategies to residents’ preferences for support, structure, and formal/informal social interaction.

## Introduction

Medical residents undergo many challenging transitions during their residency, including shifting from student to resident, changing rotations, and moving between hospitals [1,2,3,4]. With each transition, residents must adapt to new tasks and roles [5] while integrating into an established healthcare team, each with its own norms, values, and rules of interaction [5,6,7]. These frequent transitions can be stressful and have potentially negative consequences for both residents and patients [2,4]. Residents may struggle to acquire the knowledge, skills, and attitudes, which constitute the foundational elements of competence, essential to take full ownership of patient care [2,4].

This study explores residents’ transitions by employing organizational socialization, a concept rooted in organizational psychology that refers to the period during which newcomers acquire the knowledge, skills, and behaviors needed to assume their roles within an organization [8]. Organizational socialization theory posits that onboarding programs are crucial to newcomers’ socialization processes [9,10]. For health professions education, onboarding programs encompass both formal (e.g., orientation programs) and informal (e.g., interactions with other healthcare professionals) socialization strategies [7,11,12].

The onboarding programs for healthcare team members transitioning into new roles vary substantially. Some programs are comprehensive, including both formal and informal orientation elements [11]. Formal orientation programs are typically implemented by healthcare departments or hospitals and involve courses that address workplace-specific knowledge and skills; guidance from role models, mentors, or fellows; and insights into logistical aspects [5,7,11,13,14]. In contrast, informal programs involve social interactions between residents and healthcare team members (i.e., supervisors, nurses, and fellow residents) and focus on providing support and respect [5,14], fostering an open atmosphere, and tailoring the orientation to individual needs (e.g., informal guidance on work agreements, rules, protocols, how to perform duties) [11,12,15]. Despite considerable research into both formal and informal orientation programs, residents still perceive them as inadequate or absent for various reasons [2,5,13,14,15,16], including high resident-to-supervisor ratios, scheduling challenges, shift work, and service-related work environment pressures [11]. Hospitals vary widely in their onboarding approaches, as do residents’ perceptions of them [2,12]. Some residents prefer a formal, hospital-wide approach; others seek informal orientation approaches that allow them to experiment [2,12]. Given variation in residents’ preferences, can we identify types of residents group them according to their socialization preferences? To our knowledge, no research has yet identified the most beneficial strategies for specific resident types. Therefore, we aim to identify patterns in residents’ preferences for strategies to facilitate their transition. Understanding these patterns can help improve tailoring supervision and guidance to the unique needs of individual residents during their transition, which may improve their well-being and health care performance.

## Methods

### Context and setting

We conducted this study in eight hospitals within the Northeast Education Region of Postgraduate Training (OOR NO) in the Netherlands. Participants, recruited in March 2023, were residents who had completed a six-year undergraduate medical education program. They were working as either residents not in training or as first- or second-year specialty training residents [17]. In the Netherlands, residents not in training customarily work for approximately 3.5 years before commencing their postgraduate specialty training, which typically lasts three to six years [18,19]. This training takes place in both academic and general non-academic teaching hospitals. Residents not in training often spend a year working in a specific department, after which they may choose to apply for another year as a resident not in training or for a position in a specialty training program. In the Netherlands, postgraduate residents are selected by a selection committee comprising various Program Directors from the specific specialty training to which the resident applied. This process is high-stakes, based on performance-based selection criteria, and implicit social processes such as intuition, fitting in with the group, and the personal beliefs and values of the selection committee [20]. Specialty training residents rotate, depending on the program, every three to 12 months to other (sub)departments or hospitals.

### Design

We used Q-methodology to identify patterns in residents’ preferences for strategies employed by healthcare team members, departments, and hospitals to optimize their transition. Researchers have often applied Q-methodology in health professions education research to identify patterns in intention of certain behaviors or other subjective matters [21,22,23,24,25,26]. This mixed-methods research technique aims to capture individual respondents’ subjective viewpoints systematically, using preference similarities, to identify groups with similar viewpoints [27,28]. Q-methodology offers nuanced insights through the combination of qualitative and quantitative data. It identifies shared viewpoints, enhancing our understanding of subjective experiences and preferences, ultimately leading to more comprehensive and context-specific research findings [26,27]. We adhered to the steps typically followed in the development and execution of Q-methodology studies: (1) develop the statement set; (2) recruit a purposeful sample of participants; (3) have participants sort statements into a grid, which takes on the form of a quasi-normal distribution ranging from ‘not my preference at all’ to ‘totally my preference’ (Appendix 1: Figure 2); (4) ask participants to explain their sorting; (5) analyze the data using a reduction technique called ‘by-person (inverted) factor analysis’, which clusters participants instead of items, resulting in a typology of participants who share similar preferences, which capture their viewpoints [28]; and (6) identify and interpret groups with distinct viewpoints.

### Developing the statement set

We derived the statement set from prior qualitative interview studies into residents’ perceptions of organizational strategies employed by healthcare team members (e.g., supervisors, nurses, fellow residents) to optimize residents’ transition, as well as program directors’ perspectives on their roles [7,12]. The interview data proved valuable and provided insights into residents’ experiences with organizational strategies and program directors’ strategies aimed at optimizing their transition. We identified six organizational socialization tactics [7,12].

To start, one team member (GG) divided the collected statements into six tactics (collective-individual, formal-informal, sequential-random, fixed-variable, serial-disjunctive, investiture-divestiture) to ensure the statement set covered all relevant aspects [7,8,9,10,12,29], then rephrased all statements to match the research question and instructions to participants so that every phrase started with ‘*I like* …’. Three team members (GG, GW, JS) independently assessed each statement for uniformity, clarity, understandability, and suitability. We also determined whether the statements were equally distributed on the level of interaction between residents and healthcare professionals, as well as the systemic level (department/hospital), because organizational tactics influence residents’ introduction at both levels [12]. We clustered statements of comparable themes into the six tactics and combined, rephrased, or discarded overlapping statements. Each research team member reviewed the preliminary statement set to assess its clarity and understandability. Subsequently, GG piloted the preliminary statement set in individual (online) interviews with four residents, using techniques such as thinking aloud and verbal probing [30,31] by asking them to verbalize every thought while reading the statements. GG also used probe questions to assess how residents formulated statements, allowed participants to (dis)agree with the statements, and checked whether they accurately represented the topic [32]. We tested the procedure on three medical students and one resident to ensure clarity and proper execution. The pilot residents and medical students followed the same procedure as the participants, as detailed in the ‘sorting participant statements’ subsection below.

### Recruiting a purposeful sample

Typically, Q-methodology studies involve 40–60 participants [28]. To achieve maximum variability in the participant sample, we purposefully sampled participants [28]: We specifically targeted participants from three groups (residents not in training and first- and second-year specialty training residents) to address the transition experiences of early-career doctors. With a deliberate approach, we also systematically included residents from each of the three hospital-based specialties (medical, supportive, and surgical) [33] and from academic and general teaching hospitals. We concluded sampling once the inclusion criteria were met.

### Sorting participant statements

**Figure 1:**
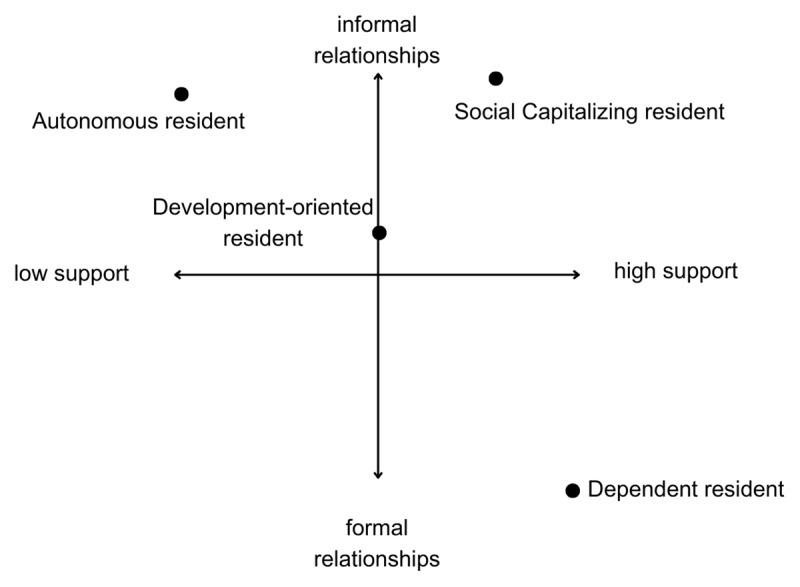
Relative Overview of Four Viewpoints on Two-Dimensional Scale. *Notes:* The x-axis represents preference for the degree of support, at the level of both supervisors and the department/hospital with a structured onboarding program. A lower position indicates a preference for minimal guidance and a loosely structured onboarding period, whereas a higher position represents a preference for substantial guidance and a highly structured onboarding period. The y-axis reflects preferences for informal or formal relationships.

We used the online tool www.qsortouch.com, as used successfully in other Q-methodology research [23,24]. Participants first gave informed consent, then received the prompting question, ‘What are your preferences regarding strategies other healthcare professionals, departments, or hospitals should use to optimize your next transition to a new workplace?’ Subsequently, they randomly read the statements and categorized them into one of three piles: ‘not my preference at all’, ‘neutral’, and ‘totally my preference’. This process allowed them to become familiar with the statements. After sorting all the statements, they refined their three piles by ranking the statements into a Q-sort grid (Appendix 1: Figure 1) with nine columns ranging from ‘not my preference at all’ (–4) to ‘totally my preference’ (+4) [28]. The rows were quasi-normally distributed, ranging from one statement in the extremes (–4, +4) to eight statements in the neutral (0). The sorting procedure ended when all statements had been placed in the fixed distribution.

### Explaining participants’ ranking

Next, participants elucidated the reasoning behind their sorting decisions and provided sociodemographic information through a post-sort questionnaire (Appendix 2).

### By-person (inverted) factor analysis

We employed a by-person (inverted) factor analysis and identified distinct factors using dedicated software (PQMethod, version 2.35) [34]. For the sake of clarity, we replace ‘factor’ with ‘viewpoint’. In Q-methodology, participants serve as variables, unlike in most quantitative research methods. Consequently, the correlation matrix reflects connections across Q-sort patterns, representing participants’ viewpoints. After considering various extraction techniques, we chose the centroid Brown option followed by a Varimax rotation (Appendix 2) [28]. With principal component analysis, we also checked for comparable results, which it supported. Within the centroid Brown–Varimax option, we considered three-, four-, and five-factor solutions. For each solution, we examined both the variance and the number of Q-sorts that loaded significantly on the extracted viewpoints. A cumulative variance of 35%–40% or higher indicated a robust solution [28].

### Identifying and interpreting distinct viewpoint groups

We employed three decision-making criteria to determine the number of viewpoints. First, we used the Kaiser-Guttman rule to retain viewpoints with an eigenvalue of at least 1, though this rule often overestimates the number of viewpoints [28,32]. Second, we accepted viewpoints that at least three participants loaded on significantly (*p* < .01), which corresponded to a loading greater than .40 (i.e., 2.58 × [1/√number of items in the Q set]) [28,35]. Third, we examined the standard error, which corresponded to.15, calculated using 1/√number of items in the Q set. Using Humphrey’s rule (i.e., to count as a viewpoint, the cross-product of its two highest loadings should exceed the standard error) [28], we found three viable solutions comprising three to five viewpoints.

To interpret the results, we examined factor arrays of the three-, four-, and five-viewpoint solutions. A factor array represents weighted averages of the Q-sorts loading on a specific viewpoint, by describing an ‘idealized Q-sort’. Six researchers independently interpreted the solutions, examining the relative location of the statements between the viewpoints (highest and lowest ranks of statements compared with other viewpoints), as well as ‘distinguishing’ and ‘consensus’ statements [28]. A statement is distinguishing if its ranking differs significantly (*p* < .05) from its rankings in other viewpoints [32] and consensus if the ranking does not differ between any pair of viewpoints (i.e., all the study participants included in the viewpoints ranked or valued the statement in [almost] the same positive, negative, or neutral way; [28,32]). To develop a genuinely holistic interpretation of each viewpoint, we developed a crib sheet for each viewpoint. A crib sheet is used to systematically identify the highest and lowest-ranked statements, in combination with statements ranked higher or lower in the specific viewpoint than in other viewpoints [28]. We used the post-sort questionnaire to understand the perspectives expressed by each viewpoint, in that answers to the open questions provided information about why residents chose statements at the extremes (–4, +4) (Appendix 2).

## Results

The initial statement set consisted of 162 statements, which we reduced to 42 (Table 1). We purposively selected 52 participants (Table 2) and excluded 1 who did not complete the post-sort questionnaire. Our centroid Brown–Varimax analyses identified four viewpoints, as explained in Appendix 3. In this solution, 36 of the 51 participants loaded on one of the viewpoints (71%), which corresponds with a suitable 51% variance [27]. The remaining 15 participants were either non-significant (n = 5) (i.e., not loading significantly on any of the viewpoints) or confounded (n = 10) (i.e., loading significantly on more than one viewpoint). We further identified three consensus statements (italicized in Table 1), representing relative shared agreement (Appendix 4).

**Table 1:** Factor (viewpoint) Array, complete list of 42 statements and idealized sorts for the four viewpoints representing residents’ preferences for onboarding strategies.


	STATEMENT VIEWPOINT	1	2	3	4

1	I like it when we as residents actively form a cohesive group ourselves, without support from the program director or department.	–1*	1	1	**2**

2	I like it when supervisors and nurses treat me like one of (many) residents.	–2	–1*	–2	–2

3	I like it when the department takes the initiatives to help us as residents become a cohesive group.	–1	0	0	–1

4	I like it when my colleagues^ actively invite me to be part of the care team, for example, by going out to lunch together.	–1*	3*	1	1

5	I like it when the program director and residents together monitor compliance with the collective bargaining agreement (work/life balance).	0	0	–1*	**1**

6	I like it when colleagues^ support me during tough moments, for example, a difficult collaboration.	**2**	1	0	0

7	I like to interact informally with colleagues^.	–2*	2	2	1

8	I like it when my fellow residents tell me in the first few days what the unwritten rules are in the department, such as how to approach supervisors.	**–1**	3*	1*	–1

9	I like it when my fellow residents introduce me, for example, by helping me with the department rules and daily schedule and giving suggestions on how to do the work.	1	2	2	**0**

10	I like that before I start working at the hospital I have already been sent an introduction document and additional information about the hospital.	–1	0	**1**	**–2**

11	I like it when the first few months are structured with, for example, a clear onboarding, getting to know the hospital, specific courses, and shadowing days at different places.	0	2*	–1	–1

12	I like to take courses with fellow residents.	–2*	2*	0	–1

13	I like it when there is a defined onboarding period of several weeks.	**0**	–1	–2*	–1

14	I like that I am not allowed to perform the duties of a resident until I feel familiar with the departmental procedures and working methods.	–2*	–1	–2*	**0**

15	I like it when I can explore my tasks quietly and alone during the onboarding period.	–1*	–3	–3	0*

16	I like it when there is no clear onboarding period, so that I can figure things out independently.	–3	**–4**	–1*	–3

*17*	*I like being given the space to make mistakes*.	*2*	***3***	*2*	*2*

18	I like it when the onboarding period concludes with a conversation with the program director or a supervisor about my functioning (what is going well, what could be improved).	0	**1**	0	0

19	I like it when the number of patients I am responsible for gradually increases.	1	–1*	–2*	1

20	I like being thrown in at the deep end.	–2*	–3	2*	**–4**

21	I like it when I can indicate how much work I want and have it tailored to my (performance) level.	0	0	–1	–1

22	I like to learn the work by being given responsibilities and being guided in this by my supervisor.	2	2	4*	2

23	I like guidance from the supervisor to introduce myself to other colleagues.^	0*	–2*	**–4**	–2

24	I like it when colleagues^ adapt their behavior to me as a newcomer by helping me and adjusting their expectations to my competencies.	0	0	–3*	–2

25	I like to introduce myself to other colleagues^: I do not need help from the supervisor for this.	–2	–2	1*	0*

26	I like it when it is clear in advance how long it will take before I get more tasks and responsibilities.	–1	–1	–1	1*

27	I like it when the decision of when I can do shifts or when I will get more duties and responsibilities depends on my competencies.	3*	1	0	0

28	I like it when colleagues^ show me how to perform, or how to solve a problem.	**2**	1	0	**–2**

29	I like to get space to observe other residents to better understand my role.	1*	–2	–2	–2

30	I like it when colleagues^ provide space to spar about planning the day.	1	–1	1	–1

31	I like having the space to discover for myself how to do the work (optimally).	1	–2*	2	2

32	I like to figure out for myself how each supervisor wants to be approached.	–3	–3	–1*	0*

33	I like it when I am required to conform to the norms and values of the department in order to be accepted.	–3	–2	–3	–3

34	I like to adapt to what the supervisor wants.	**–4**	–2	–2	–3

35	I like it when supervisors come immediately into the hospital during the shift when I need them.	4*	2	**1**	3

*36*	*I like it when I do not know something, I can approach the nurse easily*.	*2*	***1***	*2*	**3**

37	I like it when people who like me but also people who don’t like me help me become a better doctor by providing feedback.	2	0*	**3**	2

38	I like it when a hospital organizes ‘peer support’, that is, help from a colleague in an intense situation.	1	0	**–1**	1

39	I like it when supervisors are personally interested in me.	3	1	3	1

40	I like it when the department allows me to share my experiences from previous hospitals or departments and that people are willing to learn from them.	1	–1*	0	2*

41	I like having the space to adapt my training plan and work to my needs.	0	0	0	3*

*42*	*I like it when there is an open atmosphere: that you can easily ask the supervisor, even if it is simple question*.	*3*	*4*	*3*	*4*


Notes: This array consists of the complete list of 42 statements and idealized sorts of the four viewpoints representing residents’ preferences regarding healthcare team members’ and departments’ strategies to optimize their transition. Within each viewpoint, we highlight the highest- and lowest-ranked statements in boldface, the consensus statements in italics, and distinguishing statements with an asterisk*. A statement is distinguishing if its ranking differs significantly (*p* < .05) from its rankings in other viewpoints and consensus if the ranking does not differ between any pair of viewpoints. ^The word ‘colleague’ refers to supervisors, nurses, and fellow residents.

**Table 2:** Participant Characteristics.


	SAMPLE	VIEWPOINT 1: DEPENDENT RESIDENT	VIEWPOINT 2: SOCIAL CAPITALIZING RESIDENT	VIEWPOINT 3: AUTONOMOUSRESIDENT	VIEWPOINT 4: DEVELOPMENT-ORIENTED RESIDENT

Respondents (n =)	51	10	9	12	5

Variance (%)		13	12	15	11

Gender					

Female	32 (63%)	8 (80%)	5 (56%)	5 (42%)	4 (80%)

Male	19 (37%)	2 (20%)	4 (44%)	7 (58%)	1 (20%

Specialty					

Medical	28	6 (60%)	4 (44%)	6 (50%)	4 (80%)

Surgical	17	4 (40%)	2 (22%)	5 (42%)	1 (20%)

Supportive	6	0	3 (33%)	1 (8%)	0

Age mean (range) (years)					

Average	28.8	29.7	29.1	28.7	29.2

Range	25–37	25–37	26–33	25–32	26–32

Rank					

Resident not in training	30 (59%)	6 (60%)	4 (44%)	7 (58%)	2 (40%)

Specialty training resident	21 (41%)	4 (40%)	5 (56%)	5 (42%)	3 (60%)

Hospital type					

General, teaching	42	9	5	10	5

Academic	9	1	4	2	0


Next, we provide an overview of each viewpoint. The statements are indicated in brackets, along with their placement in the idealized sort, corresponding to the columns of the grid. We interpreted the viewpoints according to residents’ preferences and qualitative explanations, as follows: (1) Dependent resident, (2) Social Capitalizing resident, (3) Autonomous resident, or (4) Development-oriented resident. See Table 2 for participant demographics. Figure 1 illustrates the relative positioning of the four viewpoints on a two-dimensional scale.

### Viewpoint 1: Dependent resident

Viewpoint 1 accounts for 13% of the data variance, with 10 participants significantly loading on this viewpoint. This viewpoint represents residents who preferred a *task-oriented approach, clear guidance*, and *formal relationships* with colleagues. Regarding the task-oriented approach, these residents preferred their responsibilities to be based on their competencies (S27: +3). In addition, they favored receiving clear guidance from colleagues to effectively carry out their roles, resolve issues, and familiarize themselves with departmental rules and daily routines (S9: +1, S28: +2, S29: +1). As one participant mentioned, ‘*often, this will teach you useful tricks and you will be up-to-date the quickest*’ (R42). They noted that supervisors should provide more consistent guidance, and therefore, they preferred not adapting to supervisors’ idiosyncrasies (S34: –4). One resident explained: ‘*every supervisor has different standards, and I find it illogical that residents must adapt since there should also be more uniformity among supervisors so that adapting does not just come from the residents’ (R24)*. They were neutral on whether supervisors should guide them in getting acquainted with other colleagues (S23: 0), but they strongly preferred supervisors to be available immediately during on-call situations (S35: +4). One resident expressed that if supervisors are available immediately, they show that residents ‘*are not alone*’ (P15). Furthermore, they primarily valued formal relationships and had relatively limited interest in informal social aspects of their work, like interacting with colleagues (S7: –2), participating in courses together with fellows (S12: –2), or actively seeking invitations to be part of the healthcare team (S1: –1, S4: –1).

### Viewpoint 2: Social Capitalizing resident

Viewpoint 2 explains 12% of the data variance, with 9 participants loading on it. It represents residents who prioritized a *structured onboarding period* and *social interaction*. A structured onboarding period allowed them to familiarize themselves with the hospital environment, participate in courses, and observe others (S11: +2). Consequently, they did not prefer the absence of an onboarding period (S16: –4) but rather wanted it to conclude with a conversation with the program director or supervisor, allowing for personal feedback on their performance (S18: +1). As one participant emphasized: *I think personal feedback is very important. At such a time, you can discuss the rest of the year. What is going well, what could be improved, where you need to work on, and how you can prepare for on-call situations. What do [people] expect from you. (R28)*

The residents valued becoming part of the team, such as having lunch together (S4: +3) and receiving insights from fellow residents about the unwritten department rules, including how to approach different supervisors (S8: +3). They found it beneficial to attend courses together with colleagues (S12: +2).

### Viewpoint 3: Autonomous resident

Viewpoint 3 accounts for 15% of the data variance and 12 participants who load significantly on it. It represents residents who preferred a *loosely structured onboarding period, independence, responsibility*, and *social interaction*. Compared with other viewpoints, residents with this viewpoint were less concerned about the onboarding period lacking clarity and requiring them to figure things out independently (S16: –1). They preferred to explore their tasks on their own terms; the onboarding period did not need to be rigidly defined (S13: –2). They did not desire a sequential increase in the number of patients either (S19: –2). They valued learning their work by being given responsibilities (S22: +4) and being thrown into the deep end (S20: +2). They were comfortable introducing themselves to colleagues without relying on supervisor assistance (S25: +1, S23: –4). As one participant expressed, ‘*I can do that on my own, it seems to be a part of professional performance’ (R6)*. Furthermore, they saw the benefits of learning from their fellow residents. They acknowledged that their peers could help them understand unwritten rules (S8 +1) and learn how to approach different supervisors (S32: –1). They did not expect their colleagues to adapt their behavior to residents as newcomers (S24: –3); they simply wanted to be treated like everyone else. As one resident stated: ‘*I do not want to be treated differently’ (R31)*.

### Viewpoint 4: Development-oriented resident

Viewpoint 4 explains 11% of the data variance, with 5 participants loading significantly on it. It represents residents who sought an *intermediate structure*, allowing for *independence, exploration*, and *development*. These residents preferred not to be thrown in at the deep end (S20: –4). Instead, they sought a gradual increase in workload and responsibility, with prior information about department procedures, working methods, and knowledge of when to become accountable for what (S14: 0, S26: +1). They appreciated that the program director and residents shared responsibility for compliance with the collective bargaining agreement (S5: +1). However, they also desired some space for their own input: *I like to have a structure during my onboarding period, especially in a new hospital with new colleagues. Within this structure, there may be some freedom, but it should be clear where I work each week during the onboarding period. (R16)*

They did not prefer an introduction document before they started in a new department (S10: –2). Moreover, they wanted a certain level of independence. They did not desire guidance to form a group of residents (S1: +2) or support to perform tasks or solve problems (S28: –2). Furthermore, they appeared neutral about support from residents to understand department rules, daily routines, and suggestions for how to improve (S9: 0). Compared with residents with other viewpoints, these residents were less concerned about exploring their tasks independently during the onboarding period (S15: 0). They prioritized development and valued bringing previous experiences from other hospitals or departments to their current workplace (S40: +2). They also appreciated the opportunity to tailor their personal development and work to their needs (S41: +3).

## Discussion

This study’s aim was to identify and compare residents’ preferences regarding strategies employed by healthcare team members and departments to optimize residents’ transition. We identify four distinct viewpoints. Our results both confirm and build on previous studies focusing on various aspects of onboarding programs. Our results confirm previous studies by pinpointing the importance of guidance by other healthcare professionals, onboarding program structure, and social interaction methods [5,7,11,12,13,14,15]. The novel perspective our study contributes is that residents vary considerably in their preferences for supervisory guidance, onboarding program structure, and social interaction methods. This diversity of preferences suggests that a one-size-fits-all approach may be inappropriate [36] and underscores the clear need for tailored approaches to enhance transition experiences within healthcare teams and departments.

Our findings enrich the existing literature, by shedding light on factors that contribute to the perceived inadequacy or absence of formal and informal onboardings in clinical practice [2,5,13,14,15,16]. The considerable variance in residents’ preferences for how other healthcare professionals and departments facilitate their transitions—particularly regarding the levels of structure and formality and emotional bonding in social interactions—may explain why some organizational strategies work for some residents but not others. For example, a structured and formal onboarding period may appeal to and fit the preferences of Social Capitalizing, Dependent, and Development-oriented residents, but not those of Autonomous residents.

This study confirms that residents’ preferences for support in the onboarding period vary significantly, which possibly clarifies why many residents experience dissatisfaction with their onboarding period in previous research [5]. Organizational socialization research underscores the significance of aligning organizational practices with newcomers’ expectations; doing so correlates with heightened job satisfaction and retention [37] whereas unmet expectations frequently result in dissatisfaction and turnover [38]. To bolster residents’ satisfaction and retention, it is imperative for healthcare professionals to tailor their strategies to individual residents’ preferences. Understanding residents’ preferences for the level and kind of support (specifically, the amount of structure during the onboarding period and the formality and emotional bonding in social interactions) can be instrumental in achieving this goal. This approach aligns with research outcomes in health professions education that support the idea of customizing strategies to optimize the transition experiences of both students and residents [7,12,23].

Our data display the presence of four types of residents, which implies that program directors, other healthcare professionals and Postgraduate Medical Education (PGME) institutions and, last but not least the residents themselves, may benefit from tailoring the socialization strategies to individual residents’ needs. Tailoring strategies to residents’ needs also matches the central premises of self-determination theory (SDT), which identifies three basic psychological needs: autonomy (sense of choice), competence (sense of capability), and relatedness (sense of belonging) [39]. Supporting these needs fosters intrinsic motivation among residents, leading to positive outcomes like improved performance, adjustment, and positive well-being [40]. Although everyone possesses these three basic needs, their distribution varies over time, context, and individual preferences [41,42,43], and this variation in turn corresponds with fluctuations in preferences for support and social relationships during residents’ transition. Thus, we again underscore the importance of intentionally addressing residents’ needs for socialization support.

### Limitations and further research

The Q-sort methodology offers somewhat limited generalizability to settings beyond the scope of our research. Although prior studies have used the theory of organizational socialization in other transitioning settings (e.g., nurses, preclinical medical students), continued research is needed to confirm whether our results transfer to such settings [13,44,45]. In addition, Q-methodology benefits from diverse participant samples. In our study, we did not employ predetermined criteria for selecting residents with varying preferences. Consequently, we did not identify any participants who favored minimal support and formal relationships. This limitation may be attributed to our sampling strategy, where individuals who do not favor low support and formal relationships might have been less inclined to participate in our research. Alternatively, it could indicate a broader trend in resident selection in the Netherlands, where candidates ‘who fit in the group’ are typically selected [46]. Future research should involve a purposive sample of residents from different cultural backgrounds. Currently, it is known that residents with a migration background are less likely to pursue a career as a medical specialist, possibly due to socialization processes and challenges with fitting in [46,47]. Furthermore, our study only captured junior residents’ preferences at a specific point in time, whereas preferences may change over time [48]. Therefore, additional research might investigate differences in the preferences of junior and senior residents (e.g., final specialty training residents), to determine how preferences develop over time and which factors influence such developments [48].

An additional limitation is that the interpretation and choices for the factor solution depended on the research team. We recognize that a different research team might have opted for an alternative factor solution, leading to different outcomes.

### Practical implications

To align with SDT principles, healthcare departments must individualize socialization strategies, by reflecting residents’ preferences and tailoring strategies to their autonomy needs (e.g., kind and level of support, in terms of structure, formality, and emotional bonding in social interaction). Our typology of needs can be instrumental in such efforts. For example, Dependent residents primarily value support and formal relationships, so healthcare departments could encourage their competence development through constructivist learning approaches, such as a formal introduction program led by peer residents and supervisors about how these residents should do their work. For Social Capitalizing residents, both relatedness and competence are pivotal, as might be promoted through collaborative learning activities. Examples include a peer group introduction program, lunch together in which residents can discuss the unwritten rules in the department, and a structured onboarding period concluded with a conversation with the program director focused on their functioning. Conversely, Autonomous residents favor informal relationships and intentional support. In this context, autonomy takes precedence and can be nurtured through an informal introduction, opportunities to choose accountable learning activities, and reflective learning stimulating independence (such as no gradual increase in the numbers of patients but being responsible for many patients at once). Finally, Development-oriented residents seek an intermediate structure that allows for exploration, development, and independence, indicating that autonomy and competence development should be emphasized. These residents likely would benefit from not being thrown in at the deep end, but by being given clarity about the procedures and working methods in the department, and clarity about when their responsibilities will increase. To stimulate their development orientation, healthcare professionals should ask them if they have suggestions for improvement in the current work situation, based on their previous experiences in other working places. The residents should also be asked about their individual training goals, by discussing their individual training plan. To support each viewpoint, we recommend pre-transition conversations among program directors, supervisors, and residents, designed to empower residents to express their preferences and thereby enhance their autonomy, competence, and relatedness. Addressing residents’ basic psychological needs in these conversations and aligning them with one or more viewpoints not only reduces the risk of needs frustration but also can enhance residents’ motivation.

## Conclusion

We aimed to identify patterns in residents’ preferences for socialization strategies employed by healthcare teams and departments to facilitate residents’ transitions. We identified four distinct preference patterns, each with its own unique preferences for structure, formality, and emotional bonding in social relationships during the transition period. These variations underscore the need for tailored approaches to optimize residents’ transition experiences in healthcare teams and departments regarding structure, formality, and emotional bonding in social interactions. Such tailored approaches are vital for their socialization and ultimately contribute to residents’ satisfaction, retention, and overall success in healthcare settings.

## Data Accessibility Statement

The data set used and/or analyzed during the current study is (partially) accessible through the corresponding author upon reasonable request. Availability may be limited, because not all participants provided informed consent for the future use of their data.

## Additional File

The additional file for this article can be found as follows:

10.5334/pme.1189.s1 Appendices. Appendix 1–4.

## Abbreviations

GG: Gerbrich Galema, GW: Götz Wietasch, JS: Johanna Schönrock-Adema, LL: Lotte van Lieshout, IG: Ilse Grit, JB: Jonathan Bradbeer, SDT: self-determination theory.
