# Supplementary material for: Patterns of Medical Residents’ Preferences for Organizational Socialization Strategies to Facilitate Their Transitions: A Q-study
Source: Perspect Med Educ. 2024 Mar 11;13(1):169–81. doi: 10.5334/pme.1189 (PMC10941690; doi:10.5334/pme.1189)
Supplement: Appendices. — Appendix 1–4. [file pme-13-1-1189-s1.pdf]

Appendix 1.

Fig 2. The 42-statement sorting grid, with the prompting question participants used to arrange the statements.

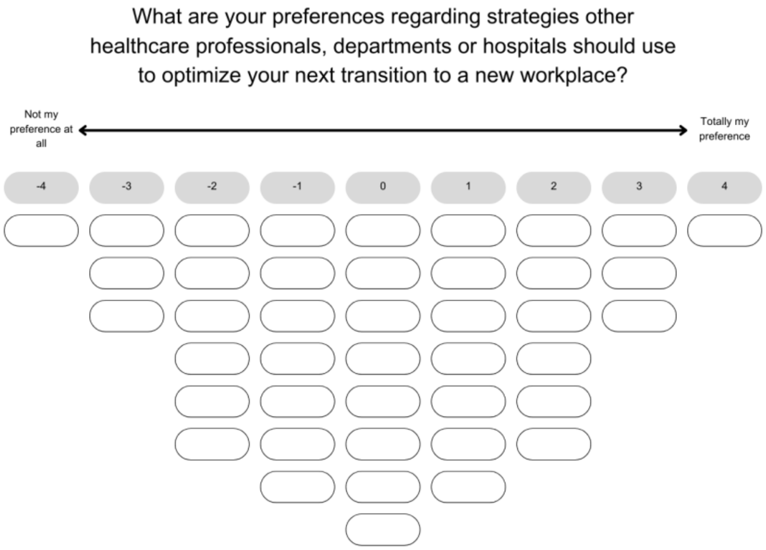

Appendix 2. Post-sort questionnaire

For proposition participant strongly did not prefer (−4):

1. Can you explain why this statement is not your preference at all?

For proposition participant preferred strongly (+4):

2. Can you explain why this statement is entirely your preference?

Demographics

3. What is your age?
4. What is your gender?
   a. Female
   b. Male
   c. Other
5. In which specialty do you work?
   a. Surgical specialty (including thoracic surgery, general surgery, neurosurgery, maxillofacial surgery, orthopedic surgery, plastic surgery, obstetrics and gynecology, ophthalmology, otorhinolaryngology, urology)_
   b. Medical specialty (including cardiology, geriatrics, internal medicine, neurology, pediatrics, psychiatry, rheumatology, dermatology, gastroenterology, rehabilitation medicine, pulmonary medicine)
   c. Supportive specialty (including microbiology, radiology and nuclear medicine, pathology, anesthesiology, clinical genetics, radiotherapy)
6. What is your experience?
   a. Resident not in training
      i. < 1 year clinical experience
      ii. > 1 year clinical experience

   b. Specialty training resident
      i. Postgraduate year 1
      ii. Postgraduate year 2
7. How many residents are working in your department (including residents not in training and residents in specialty training)?
   a. 1–5
   b. 5–10
   c. >10
8. What type of hospital do you work in?
   a. Academic
   b. General
9. Where did you study medicine?
   a. University Medical Center Groningen
   b. Amsterdam University Medical Center, location AMC
   c. Amsterdam University Medical Center, location VUMc
   d. University Medical Center Utrecht
   e. Radboud University Medical Center Nijmegen
   f. Leiden University Medical Center
   g. Erasmus Medical Center, Rotterdam
   h. Maastricht University Medical Center
   i. Other
10. In this study, we define a transition as ‘the transition from one hospital to another’. How many transitions have you experienced, counting from the time of graduation?
   a. None: I work at the same hospital where I did my final internship.
   b. 1
   c. 2

    d. 3
    e. 4
    f. >4

11. How long have you been working in your current department?
    a. 1–3 months
    b. 4–6 months
    c. 7–9 months
    d. > 10 months
12. Do you have the feeling that you know the ins and outs of the department? Why or why not?
    a. Open answer
13. How long does it take you to get settled into a department?
    a. 1 month
    b. 2–3 months
    c. 4–6 months
    d. >6 months

Appendix 3. Decision-making criteria.

On the basis of the decision-making criteria, we determined that we could extract three to five factors from the data set. We conducted a by-person factor analysis with Brown centroids for three, four, and five factors.

Table 3. Decision-making criteria: Principal component analysis (PCA) and centroid Brown methods (Watts & Stenner, 2012: 105)

Decision-making criteria:	Centroid Brown (n = number of factors to be extracted based on the specific criterion)	PCA (n = number of factors to be extracted based on the specific criterion)
Kaiser Guttman criteria	6	8
Significant loading at the 0.01 level (>0.40)<br>- At least 3 q-sorts	4	5
Humphrey’s rule: cross product of its two highest loadings exceeds the standard error (0.15)	4	5
Scree test (only for PCA)	n.a.	4

For the four-factor solution, we conducted a PCA and Centroid Brown factor array. The factors emerged as similar, indicating that the different methods did not produce significantly different outcomes.

We developed crib sheets based on the decision-making criteria for the three-, four-, and five-factor solutions (centroid-Brown–Varimax). Six researchers (GG, JS, GW, IG, JB, LL) independently interpreted the cribsheets. Then we held a meeting to discuss our findings. We came to consensus that the 5-factor solution was not appropriate; it did not meet all the decision-making criteria (i.e., one factor only had 1 significant loading at the .01 level). The three- and four-factor solutions met both the criteria (Appendix 2, Watts & Stenner, 2012: 4): They

1. Maximize the number of q sorts that load significantly on the extracted factors,

2. Explain a healthy amount of the overall study variances, and
3. Allow for satisfying both (1) and (2) using an appropriate number of factors.

Table 4, Comparison of the Three-factor and Four-factor solution

	Three-factor	Four-factor
Number of Q-sorts loading	39	36
Number of Q-sorts per factor (F1-F2-F3-F4)	17-13-9	10-9-12-5
Variance (%)	48	51
Variance per factor (F1-F2-F3-F4)	18-17-13	13-12-15-11

Although the three- and four- factor solutions were similar, in the four-factor solution, the fourth factor accounted for 11% of the variance, with five sorts loading significantly. Furthermore, this factor was distinguishable from the other three factors and easily interpretable.

Appendix 4. Consensus statements

Residents across all viewpoints expressed preference for an open atmosphere (S42, +3, +4, +3, +4) in which they had room to make mistakes (S17, +2, +3, +2, +2). In the post-sort questionnaire, residents emphasized that an open atmosphere contributed to safe patient care, job satisfaction, and a safe learning environment where they could learn from their mistakes, stating, for example:

> *Making mistakes is inevitable in a new job (or even in longer employment), but if there is no room for it, it leads to bluffing or concealment, which is detrimental both to the patient and to one's own learning process. (R43)*

Furthermore, the viewpoints did not differentiate significantly in terms of nurses' value for them. All residents found it important to consult nurses when they were uncertain (S36, +2, +1, +2, +3). As one resident acknowledged: '*Nurses have a lot of practical knowledge/knowledge of clinical picture, things you sometimes miss as a beginning resident' (R5).*
